# Cardiovascular Autonomic Control, Sleep and Health Related Quality of Life in Systemic Sclerosis

**DOI:** 10.3390/ijerph18052276

**Published:** 2021-02-25

**Authors:** Angelica Carandina, Chiara Bellocchi, Gabriel Dias Rodrigues, Lorenzo Beretta, Nicola Montano, Eleonora Tobaldini

**Affiliations:** 1 Department of Internal Medicine, Fondazione IRCCS Ca’ Granda, Ospedale Maggiore Policlinico, 20122 Milan, Italy; angelica.carandina@policlinico.mi.it (A.C.); chiara.bellocchi@unimi.it (C.B.); lorenzo.beretta@policlinico.mi.it (L.B.); nicola.montano@unimi.it (N.M.); 2 Department of Clinical Sciences and Community Health, University of Milan, 20122 Milan, Italy; 3 Scleroderma Unit, Referral Center for Systemic Autoimmune Diseases, Fondazione IRCCS Ca’ Granda, Ospedale Maggiore Policlinico, 20122 Milan, Italy; 4 Department of Physiology and Pharmacology, Biomedical Institute, Fluminense Federal University, Niterói 24210-130, Brazil; gabrieldias@id.uff.br

**Keywords:** Systemic sclerosis, autonomic nervous system, heart rate variability, health related quality of life, chronic pain, sleep, depression, sympathetic

## Abstract

Chronic pain and dysautonomic symptoms deteriorate Systemic sclerosis (SSc) patients’ health-related quality of life with serious repercussions on social life and even on sleep. Heart Rate Variability (HRV) analysis can identify cardiovascular autonomic control impairment in subclinical condition. The aim of the present observational cross-sectional study was to assess the relationship between dysautonomic symptoms, quality of life status and cardiovascular autonomic profile. ECG and respiration were recorded at rest in 20 SSc patients. HRV analysis was performed using two different approaches: Linear spectral analysis and non-linear symbolic analysis. Pain was evaluated using the Numeric Rating Scale (NRS) and 3 questionnaires were administered for the evaluation of sleep quality (PSQI), mood tone (PHQ-9) and disability (HAQ). We found that sleep impairment was related to sympathetic predominance at rest measured as low-frequency/high-frequency ratio (LF/HF) (r = 0.48 and *p* = 0.033); poorer sleep quality was related to higher pain values (r = 0.48 and *p* = 0.034) and depressive symptoms (r = 0.82 and *p* < 0.01); higher pain scores were related to higher cardiovascular vagal modulation and higher disability indexes (r = 0.47 and *p* = 0.038 & r = 0.55 and *p* = 0.012, respectively). In conclusion dysautonomia and chronic pain showed a severe impact on sleep quality and disability with a consequent worsening of depressive symptom in our cohort of SSc patients.

## 1. Introduction

Systemic sclerosis (SSc) is a systemic autoimmune disease that affects both skin and internal organs characterized by microvascular involvement, autoantibody production, extracellular matrix remodeling and collagen deposition leading to fibrosis. From the most recent epidemiological data, dating back to 2016, SSc prevalence in Italy is estimated to be 306.1 per million, with an overall female to male (F/M) ratio of 7.8:1 [1]. Among autoimmune disorders, SSc is one with the worst impact on life activities due complications such as digital ulcers, skin fibrosis, tendon retractions and arthritis [2,3]. Limitations in physical functions due to pain, sleep disturbance and fatigue strongly compromise SSc quality of life with consequent depression and reduction of social activities [4,5]. Moreover, several studies highlighted the serious repercussions of chronic pain in adults patients on daily activities, work ability and mood, thus, underlining the importance of pain control interventions and their impact on socio-economic aspects and healthcare utilization [6,7]. To date, there is no effective treatment to reverse damage accrual and disease progression in SSc. Therefore, the attempt to control the disability due to disease progression is a challenge, in order to ameliorate SSc patient’s health-related quality of life (HR-QoL).

Chronic pain is known to be strictly interrelated with autonomous nervous system (ANS) dysfunction being both its trigger and its consequence [8]. Therefore, not only chronic pain, but also dysautonomic symptoms, such as esophageal dysmotility, diarrhea, occlusive syndrome and altered cardiovascular autonomic control deteriorate SSc patients’ HR-QoL [9,10]. Recently, cardiac autonomic impairment in SSc was related to the fibro-vascular progression of the disease [11]. Furthermore, several studies reported the early occurrence of autonomic dysfunctions, even before the development of fibrosis and cardiac structural changes [12,13].

Heart rate variability (HRV) is a non-invasive measure of ANS modulation on the cardiovascular system and investigations show its association at rest with cardiovascular risk and mortality in SSc [13,14,15]. In particular, the use of non-linear symbolic HRV analysis is an innovative approach in assessing non-reciprocal variations of the two autonomous branches and could represent a useful and sensitive tool in prognosis and risk stratification in SSc patients [11,16]. Moreover, HRV indexes alterations that reflect ANS dysfunction, are associated with chronic pain conditions and therapeutic approaches using vagal nerve stimulation have been attempted in several chronic conditions, in which pain is a key feature, such as refractory migraine, rheumatoid arthritis and depression.

On these premises, the present study was undertaken to test the hypothesis that, in a cohort of SSc patients, HRV indexes are associated with SSc HR-QoL assessed by validated scales and questionnaires.

## 2. Materials and Methods

### 2.1. Study’s Design and Population

For the present monocentric cross-sectional study, we enrolled all SSc patients (N = 20) who were experiencing pain and who consented to participate in the study from the Day Hospital of Internal Medicine, Immunology and Allergology Department, (Fondazione IRCCS Ca’ Granda, Ospedale Maggiore Policlinico, Milan, Italy). All the patients fulfilled the 2013 American College of Rheumatology/European league against rheumatism classification criteria for SSc [17]. Patients included were experiencing pain, assessed by Numeric Rating Scale (NRS), over the seven days prior to enrollment. The absence of a stable sinus rhythm on the ECG, ongoing therapy with beta-blocker drugs, pregnancy and consent refusal were considered exclusion criteria for this study.

The enrollment and recording period elapsed between March 2019 and January 2020. Every patient underwent a single assessment: Each experimental session consisted of a resting ECG and respiratory recording, pain assessment and the administration of 3 questionnaires. All experimental sessions took place between 8 a.m. and 12 noon. The protocol was approved by the local Ethics Committee (Comitato Etico Milano Area 2: 158_2019bis) and it was developed in accordance with the Declaration of Helsinki. All the subjects signed informed written consent before participation to the study.

### 2.2. Physiological Recordings

Cardiovascular recordings were performed at rest, in the supine position and with spontaneous breathing for 10 min. All participants were informed to avoid taking food and caffeine in the 2 h preceding the recording session and physical exercise the day before. ECG (lead II) and respiration through a thoracic piezoelectric belt were recorded with a sampling frequency of 250 Hz, using an ad hoc telemetric system device. All measurements were performed in a quiet and temperature-controlled room (between 22 °C and 24 °C), and all patients had a normal body temperature during the recordings (between 35.5 °C and 36.5 °C).

### 2.3. Pain and Health-Related Quality of Life Assessment

Subjects were asked to report the pain perceived in the week preceding the recording session on NRS with numbers from 0 to 10 (‘no pain’ to ‘worst pain imaginable’). NRS cut-offs are ≤4, 5–7, and ≥8 for mild, moderate, and severe pain, respectively [18]. Functional disability was evaluated by the Health Assessment Questionnaire (HAQ), the score range is between 0 and 3 and HAQ scores >1 are considered to indicate the presence of disability [19]. The Patient Health Questionnaire-9 (PHQ-9) was administrated for the evaluation of depressive symptoms, the PHQ-9 score can range from 0 to 27 and a PHQ-9 score ≥10 had a sensitivity of 88% and a specificity of 88% for major depression [20]. Finally, subjects were asked to report the sleep quality through the questionnaire Pittsburgh Sleep Quality Index (PSQI). In particular, we considered the global score of the test, which has a possible range of 0–21 points, and the total score of the Sleep Disturbances category represented by item 5. A global score higher than 5 is considered as an indicator of relevant sleep disturbances [21].

### 2.4. Cardiovascular Autonomic Control Assessment

Segments around 300 consecutive beats were selected from the ECG signal for the analysis of HRV. Two different approaches, linear spectral analysis and non-linear symbolic analysis, were applied through a specific software (Heart Scope II, AMPS, ITA). The autoregressive model was performed to identify the spectral power in the low-frequency band (LF, bounded between 0.04 and 0.15 Hz), an index of sympathetic modulation and baroceptive activity, and in the high-frequency band (HF, bounded between 0.15 and 0.40 Hz), an index of parasympathetic modulation and synchronous with respiration. The LF and HF components were expressed in absolute values (ms^2^) and normalized units (LFnu and HFnu) to represent the relative amount of each component compared to the total power of the HRV spectrum. The algorithm also calculates the LF/HF ratio, which is considered an index of the sympatho-vagal balance [22].

The spectrum of respiratory activity comprises a principal component, RESP HF, whose central frequency, in physiological conditions, is very close to that of the heart rate HF band. Using the autoregressive analysis, K^2^RR-RESP measures the coherence between the respiratory oscillation and the cardiac cycle and is a marker of cardiopulmonary coupling. Values range from 0 to 1, with higher values indicating a stronger coupling between heart period and respiratory oscillations [22]. The power of RESP HF and the maximum coherence at HF bands was calculated.

Nonlinear inter-beat dynamics were evaluated on the same segments by symbolic analysis. The R-R time series was converted into a sequence of symbols that was divided into 3-beat patterns. Patterns were classified into 4 families: (a) 0V, patterns with no variation, all 3 symbols are equal; (b) 1V, patterns with 1 variation, 2 consecutive symbols are equal forming a 2-beat plateau, while the remaining one is different; (c) 2LV, patterns with 2 like variations, all symbols are different from the previous one and they are in ascending or descending order; (d) 2UV, patterns with 2 unlike variations, all symbols are different from the previous one but not in a consequent order. The percentage of the patterns 0V is a marker of cardiac sympathetic modulation and 2UV or 2LV are markers of cardiac vagal modulation [23]. With respect to spectral analysis, this approach was found suitable to assess non-reciprocal changes in of sympathetic and parasympathetic modulation on heart period time series both in physiological and pathological conditions, especially those characterized by low global variability [24,25]. Furthermore, as it is focused on short patterns in the RR interval series, this type of analysis is more suitable for the study of short non-linear heart rate variability instabilities.

### 2.5. Statistical Analysis

Data were analyzed using SigmaStat software (2016 Systat Software, Inc., Chicago, IL, USA). Results were expressed as the median and interquartile range (25°–75° IQR). The Shapiro-Wilk test was used to evaluate the normality of data. The Pearson Product Moment Correlation was applied to explore the association between cardiovascular autonomic control variables and HR-QoL scores and among the results of questionnaires. *p* < 0.05 was considered statistically significant.

## 3. Results

### 3.1. Demographic, Clinical and Cardiovascular Characteristics of the Study Population

A total of 20 SSc patients were included in the study. Demographic and clinical characteristics are described in Table 1. The study group was predominantly composed of women (80%). Sixteen patients had a limited cutaneous SSc (lcSSc) and 4 patients a diffuse cutaneous (dcSSc) subset. All patients experienced pain with a predominance of articular pain. The ECG recordings were performed in all 20 subjects and the median values of cardiovascular indexes at rest are shown in Table 2. All patients completed the administered questionnaires and the median values of questionnaires are reported in Table 2.

Nine SSc patients experienced severe pain, 8 SSc patients had moderate pain and 3 patients reported mild pain. Fifteen out of 20 subjects reported a significant disability in daily activities (HAQ disability index ≥1) and 9 out of 20 passed the cut-off score of PHQ-9. Finally, 18 out of 20 patients had sleep disturbances.

### 3.2. Correlation Analysis

The correlation coefficients *r* and the significance values *p* resulting from the comparisons between the study indices are shown in Table 3.

**Figure 1:**
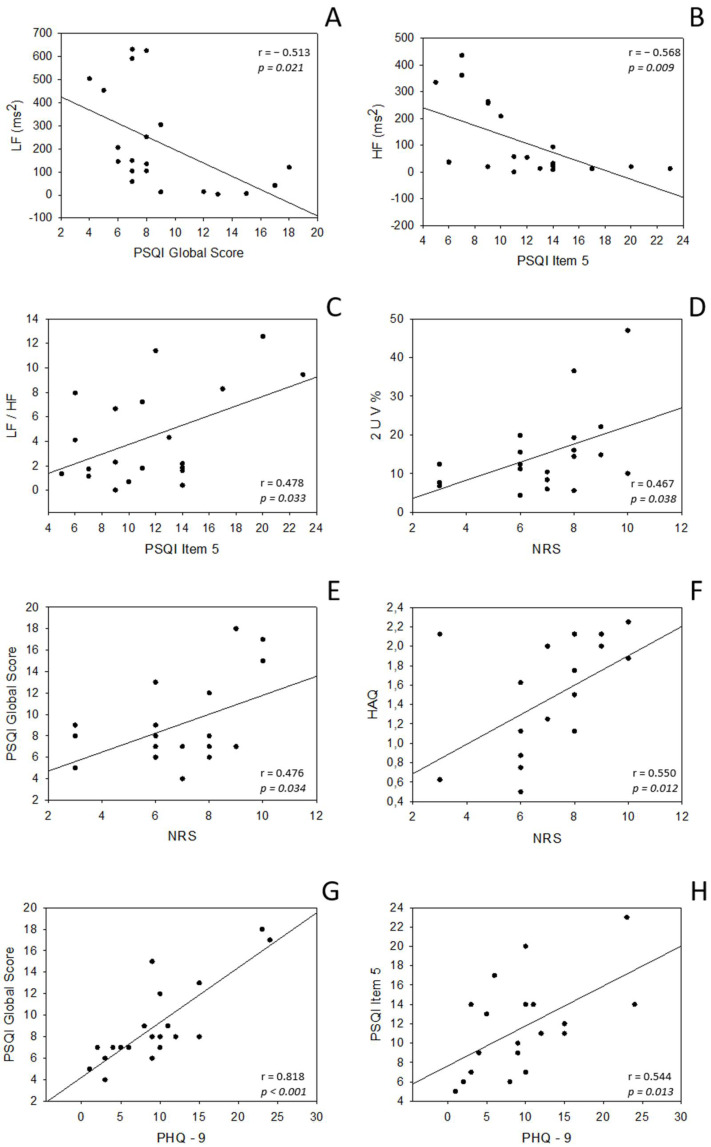
Correlation between cardiovascular parameters and questionnaires scores. Abbreviations: HF, high-frequency band; LF, low-frequency band; nu, normalized units; 2UV%, patterns with 2 unlike variations; NRS, pain score on Numeric Rating Scale; PHQ-9, Patient Health Questionnaire-9 for depressive symptoms; HAQ, index of disability assessed through Health Assessment Questionnaire; PSQI, Pittsburgh Sleep Quality Index. Significant *p* values < 0.05.

The correlation analysis highlighted a significant negative association between the global score of PSQI and LF band power expressed by absolute values (Figure 1A). Moreover, the score of Sleep Disturbances category (item 5 of PSQI) was negatively correlated with the HF band power expressed by absolute values (Figure 1B).

A positive association between the score of Sleep Disturbances category (item 5 of PSQI) and the LF/HF index was found, as reported in Figure 1C.

Surprisingly, a significant positive correlation was found between NRS pain values and 2UV%, which is an index of cardiovascular vagal modulation (Figure 1D).

Positive associations were also found between questionnaires scores. Assessment of pain through NRS positively correlated with the PSQI (Figure 1E) and the HAQ disability index (Figure 1F). The PSQI correlated positively not only with NRS pain values but also with depressive symptoms, assessed through the PHQ-9 (Figure 1G). This association was also confirmed by the positive correlation between total score of PSQI Sleep Disturbances category and PHQ-9 score (Figure 1H).

**Figure 2:**
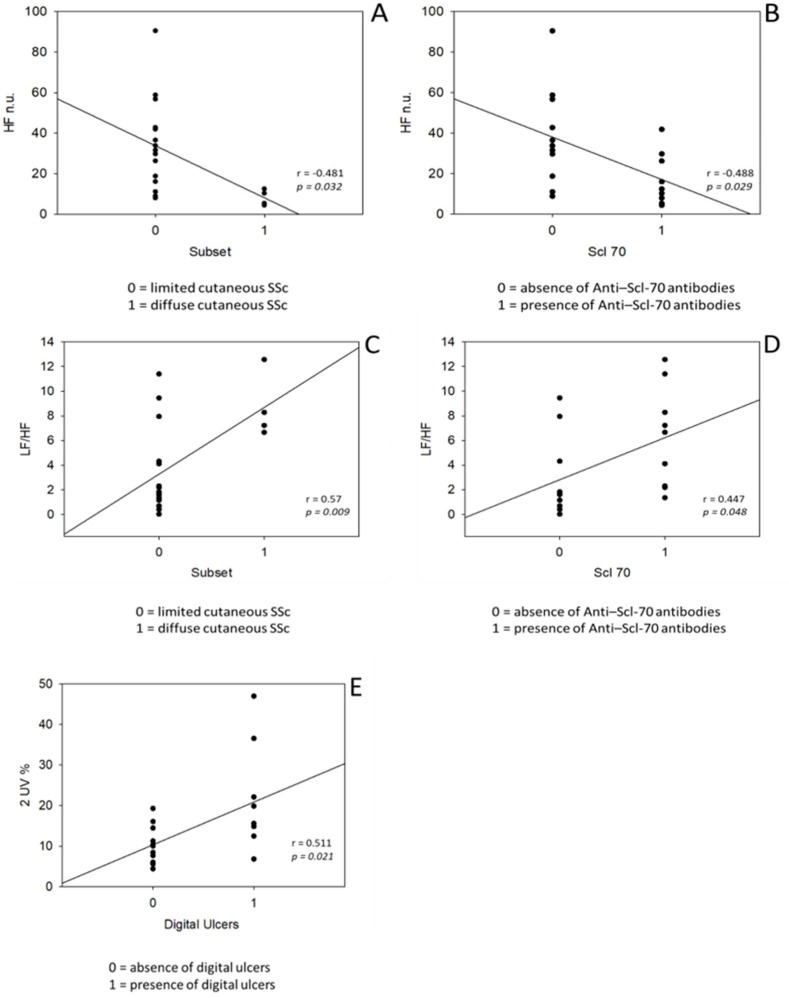
Correlation between cardiovascular parameters and SSc clinical features. Abbreviations: HF, high-frequency band; LF, low-frequency band; n.u., normalized units; SSc, systemic sclerosis; Scl-70, Anti-topoisomerase I antibodies; 2UV%, patterns with 2 unlike variations. Significant *p* values < 0.05.

We also investigated the correlation between cardiovascular parameters and SSc clinical features. Specifically, an inverse correlation between HF nu (an index of parasympathetic modulation) was observed with diffuse cutaneous subset and with anti Scl-70 antibodies positivity (r = −0.48, *p* value = 0.032; r = −0.49, *p* value = 0.029) (Figure 2A,B). The LF/HF index of sympatho-vagal balance directly correlated with dcSSc subset and with anti Scl70 positivity showing again a predominance of sympathetic response within the diffuse subset compared to lcSSc (r = 0.57, *p* = 0.009 and r = 0.48, *p* = 0.049) see Figure 2C,D. Digital ulcers presence directly correlated with 2UV% a parameter of parasympathetic modulation (r = 0.51, *p* = 0.021) (Figure 2E).

## 4. Discussion

The aim of the present study was to assess the quality of life and the association with the cardiovascular autonomic control in SSc patients with chronic pain. The major findings of this study, include that, in this population of SSc patients: (i) Sleep impairment is related to lower spectral power of LF and HF band and to a sympathetic predominance in the cardiovascular autonomic control at rest; (ii) poorer sleep quality is related to higher pain values and depressive symptoms; (iii) higher pain scores are related to higher cardiovascular vagal modulation; (iv) higher pain values are related to higher disability indexes; (v) a sympathetic predominance is correlated with the diffuse cutaneous subset and with anti Scl-70 autoantibodies positivity while the presence of digital ulcers correlates with a parasympathetic modulation.

Our results showed that alterations of the cardiovascular autonomic control in SSc are related to sleep disturbances as highlighted by the two inverse correlations between the spectral power of LF and HF band and PSQI scores. Moreover, the moderate direct correlation between LF/HF and PSQI Item 5 scores highlighted a relationship between sympathetic predominance and sleep impairment. Consistently with our observation, it is known that the autonomic nervous system modulates the cardiovascular functions during sleep onset and the transition into different sleep stages [26]. In turn, sleep disturbance has repercussions on autonomic cardiovascular control, as demonstrated by studies on acute sleep deprivation where a shift of the cardiovascular sympatho-vagal balance towards a sympathetic predominance has been observed [27,28,29]. Moreover, previous studies demonstrated the presence of an autonomic dysfunction specifically in SSc patients during the wake phase. An impairment of cardiovascular modulation has been widely described through both classical autonomic test such as tilt test, Valsalva maneuver, deep breathing, handgrip and through heart rate variability assessments [11,30,31,32]. The present data on the cardiovascular autonomic profile of SSc patients (Table 2) reflect the results of our previous study [11]. In particular, it can be seen from both studies that SSc patients are characterized by lower spectral variability, by a reduced vagal and by an increased sympathetic modulation at rest with a blunted autonomic response to physiological stimuli (e.g., the transition from supine to orthostatic position), as evidenced by both spectral and symbolic analysis.

In our study, 18 out of 20 patients reported sleep disturbances (PSQI global score >5) and we observed a positive association between sleep impairment and depressive symptoms. Sleep impairment is common in SSc, with 76% of patients reporting difficulty in sleeping and 59% reporting a moderate to severe impact of sleep lack on their daily functions [4,5]. Moreover, under these observations, previous studies identified gastrointestinal symptoms, dyspnea, pruritus and pain as determinants of a poor sleep quality in SSc [33,34]. All of these aspects of the disease have detrimental effects on daily function, especially pain, and contribute significantly in reducing quality of life.

We investigated on cardiovascular parameters correlation with SSc clinical features showing how the diffuse cutaneous subset and the anti-Scl-70 positivity are related with a sympathetic predominance. This data confirms our previous study in which we observed that these alterations are detectable mostly in the advanced and fibrotic forms of SSc.

Surprisingly, we also observed that patients who reported higher pain values showed a higher parasympathetic modulation than patients who have lower pain levels, still maintaining an overall sympathetic predominance. The literature evidence suggests that the experience of pain is inversely associated with vagally mediated heart rate variability [8,35,36]. However, most of the comparisons were conducted on healthy subjects not considering a stratification for pain chronicity. As a matter of fact, there is evidence that the negative correlation between pain intensity and vagal modulation is no longer present in those subjects that report chronic pain [35]. In their cross-sectional study, Santos-de-Araújo et al. recruited subjects with chronic neck pain identified as a Neck Disability Index (NDI) score of ≥5 points and a NRS score of ≥3 points at rest or during active cervical movement. They observed a positive correlation (r = 0.388, *p* < 0.05) between NRS value at rest and the power of high frequency band (HF nu) of HRV spectrum, a vagal index [37]. Additionally, in the present study we found also that the presence of digital ulcers correlated with a parasympathetic modulation (2UV%), in line with Gigante et al. who found that in SSc patients the parasympathetic activity was positively correlated with serum levels of vascular endothelial growth factor and disease duration [38]. Based on these observations, we can suppose that, in the long term, adaptations occur in the autonomic nervous system aimed at counteracting the pathophysiological effects of SSc.

There is a threefold connection between the autonomic nervous system, nociception and inflammation. Nociception and autonomic nervous system share many structures of the central nervous system for reception of stimuli, integration and processing of responses [39,40]. As data from neuroimaging studies revealed that chronic pain determines anatomical and functional modifications in the aforementioned areas and in descending nociceptive inhibitory pathways [41,42], we can suppose that the top-down inhibition of parasympathetic activity also fails in patients with high levels of pain, probably due to a phenomenon of habituation. Alternatively, our observations on vagal modulation may be also related to a long-term compensatory mechanism aimed at attenuating the systemic inflammation and the chronic tissue hypoxia resulting from the progressive derangement of microvasculature. As a matter of fact, findings of an increased cardiac parasympathetic modulation have been previously reported in diseases with similar inflammatory background, such as allergic rhinitis and atopic dermatitis [43,44], and concerning pro-angiogenic factors in late stage of SSc [38].

Our study has some limitations. We investigated a numerically limited cohort of 20 SSc patients with a prevalence of females, although this reflects the natural higher prevalence of Systemic sclerosis in female population. To conduct our study, we focused on a selected group of SSc with chronic pain features and mild to high pain as inclusion criteria to guarantee the homogeneity of the study population. Further studies are needed to enlarge the sample size and to investigate also patients in the early stages of the disease and with shorter disease duration.

Despite limitations, our study elucidated the interconnection between autonomic dysfunction and quality of life in Systemic sclerosis patients, investigating sleep, depressive symptoms, pain and disability with validated scales. An intriguing observation emerged from our analysis, suggesting that pain, when chronic, is related to vagal responses. Further studies will be needed to enlarge the analysis and investigate this phenomenon as a possible time-dependent adaptive response in SSc patients, as well as in other conditions characterised by chronic pain.

## 5. Conclusions

In conclusion, sleep impairment, pain and depressive symptoms are present and interrelated in patients with Systemic sclerosis, and lead to poor quality of life. In particular, sleep disturbances are correlated with a sympathetic cardiovascular predominance at rest, while chronic pain seems to lead to vagal responses as a possible adaptive mechanism in patients with higher pain values. In a chronic disease, such as SSc, sleep impairment and chronic pain should get the adequate attention from health care providers during the clinical assessment with an early recognition in order to improve patient’s quality of life.

## Figures and Tables

**Table 1:** Demographic and clinical features of SSc patients.

Characteristics	Median (IQR), *N* (%)
**Age (years)**	56 (50–65)
**Females *n*, (%)**	16 (80%)
**BMI (kg/m^2^)**	24 (23–26)
**Disease Duration (years)**	13 (10–22)
**Disease Subset**	
- lcSSc	16 (80%)
- dcSSc	4 (20%)
**Antibodies**	
- ANA+	18 (90%)
- Scl70+	9 (45%)
- ACA+	9 (45%)
**Digital ulcers**	9 (45%)
**Pain Type**	
- Articular	9 (45%)
- Myalgia	7 (35%)
- Ulcers	4 (20%)
- Raynaud	4 (20%)
- Migraine	3 (15%)

Abbreviations: SSc, systemic sclerosis; BMI, body mass index; lcSSc, limited cutaneous SSc; dcSSc, diffuse cutaneous SSc; Scl-70+, Anti-topoisomerase I antibodies positive; ANA+, anti-nuclear antibodies positive; ACA+, Anti-centromere antibodies positive.

**Table 2:** Cardiovascular and respiratory parameters and questionnaire scores of the study population.

Index	Median (IQR)
**Cardiovascular Parameters**	
**sBP (mmHg)**	120 (109–140)
**dBP (mmHg)**	75 (70–80)
**HR (bpm)**	78 (70–86)
**Spectral Analysis**	
- TP (ms^2^)	471 (198–863)
- LF (ms^2^)	140 (54–341)
- HF (ms^2^)	37 (18–221)
- LF nu	59 (49–81)
- HF nu	28 (11–38)
- LF/HF	2.24 (1.55–7.40)
**Symbolic Analysis**	
- 0V%	33 (27–44)
- 2LV%	5 (2–8)
- 2UV%	12 (8–17)
**Respiratory Parameters**	
**RESP HF (Hz)**	0.27 (0.24–0.29)
**K^2^ RR-RESP**	0.34 (0.21–0.75)
**Questionnaires**	
**NRS**	7 (6–8)
**HAQ**	1.38 (1.06–2.00)
**PHQ-9**	9 (5–11)
***PSQI***	
**PSQI total score**	8 (7–10)
**PSQI item 5 score**	11 (9–14)

Abbreviations: sBP, systolic blood pressure; dBP, diastolic blood pressure; HR, heart rate; TP, total power; HF, high-frequency band; LF, low-frequency band; nu, normalized units; 0 V%, patterns with no variations; 2LV%, patterns with 2 like variations; 2UV%, patterns with 2 unlike variations; RESP HF, central frequency of respiratory activity; K2 RR-RESP, cardiopulmonary coupling index; NRS, pain score on Numeric Rating Scale; PHQ-9, Patient Health Questionnaire-9 for depressive symptoms; HAQ, index of disability assessed through Health Assessment Questionnaire; PSQI, Pittsburgh Sleep Quality Index.

**Table 3:** Correlation between cardiovascular parameters and questionnaires scores.

Variables	NRS	PHQ-9	HAQ	PSQI Global Score	PSQI Item 5
HR	r = −0.029 *p = 0.904*	r = 0.165 *p = 0.488*	r = 0.204 *p = 0.389*	r = 0.310 *p = 0.184*	r = 0.263 *p = 0.263*
HRV Spectral Analysis					
LF ms^2^	r = −0.239 *p = 0.310*	r = −0.287 *p = 0.220*	r = −0.146 *p = 0.538*	**r = −0.513** p = 0.021 *	r = −0.392 *p = 0.088*
HF ms^2^	r = −0.028 *p = 0.907*	r = −0.427 *p = 0.061*	r = 0.214 *p = 0.365*	r = −0.401 *p = 0.080*	**r = −0.568** p = 0.009 *
LF nu	r = −0.329 *p = 0.156*	r = 0.039 *p = 0.870*	r = −0.368 *p = 0.110*	r = −0.191 *p = 0.420*	r = 0.123 *p = 0.606*
HF nu	r = 0.223 *p = 0.345*	r = −0.162 *p = 0.494*	r = 0.167 *p = 0.481*	r = 0.024 *p = 0.919*	r = −0.333 *p = 0.151*
LF/HF	r = −0.171 *p = 0.471*	r = 0.289 *p = 0.217*	r = −0.231 *p = 0.328*	r = 0.123 *p = 0.606*	**r = 0.478** p = 0.033 *
HRV Symbolic Analysis					
0V%	r = −0.222 *p = 0.346*	r = 0.081 *p = 0.735*	r = −0.296 *p = 0.205*	r = −0.158 *p = 0.506*	r = 0.087 *p = 0.716*
2LV%	r = −0.131 *p = 0.581*	r = −0.185 *p = 0.436*	r = 0.145 *p = 0.541*	r = −0.083 *p = 0.730*	r = −0.301 *p = 0.197*
2UV%	**r = 0.467** p = 0.038 *	r = −0.024 *p = 0.922*	r = 0.425 *p = 0.062*	r = 0.294 *p = 0.208*	r = 0.106 *p = 0.657*
Questionnaires					
NRS		r = 0.345 *p = 0.137*	**r = 0.550** p = 0.012 *	**r = 0.476** p = 0.034 *	r = 0.402 *p = 0.079*
PHQ-9			r = 0.153 *p = 0.521*	**r = 0.818** p < 0.001 *	**r = 0.544** p = 0.013 *
HAQ				r = 0.418 *p = 0.067*	r = 0.134 *p = 0.573*

Abbreviations: HR, heart rate; HF, high-frequency band; LF, low-frequency band; nu, normalized units; 0 V%, patterns with no variations; 2LV%, patterns with 2 like variations; 2UV%, patterns with 2 unlike variations; NRS, pain score on Numeric Rating Scale; PHQ-9, Patient Health Questionnaire-9 for depressive symptoms; HAQ, index of disability assessed through Health Assessment Questionnaire; PSQI, Pittsburgh Sleep Quality Index. In bold * Significant *p* values < 0.05.

## Data Availability

The data presented in this study are available on request from the corresponding author.
